# It’s all metacognitive: The relationship between informal learning and self-regulated learning in the workplace

**DOI:** 10.1371/journal.pone.0286065

**Published:** 2023-05-23

**Authors:** Anne Frieda Doris Kittel, Tina Seufert

**Affiliations:** Institute for Psychology and Pedagogy, Ulm University, Ulm, Germany; University of Exeter, UNITED KINGDOM

## Abstract

Informal workplace learning accounts for a large extent of employees’ learning. Informal learning activities such as reflection or keeping up-to-date resemble self-regulated learning strategies that indicate the ability to plan, monitor, and regulate one’s learning. However, little is known about the relationship between informal learning behaviors and self-regulated learning strategies. Structural equation modeling with data from 248 employees revealed that the informal learning behaviors of reflection, keeping up-to-date, feedback-seeking, and knowledge-sharing are strongly related to the metacognitive self-regulated learning strategies of monitoring and regulation. However, informal learning behaviors lack the deep-processing strategies of elaboration and organization, and the resource strategies of help-seeking and effort regulation. Only innovative behavior is strongly related to effort regulation. These results suggest a potential deficit in employees’ strategy use. Employees should consider further resources to increase their learning effectiveness in the workplace. The results are discussed, and practical implications are outlined.

## Introduction

Employees face a growing need to adapt to the rapid changes in the workplace caused by digitalization [1]. Informal workplace learning constitutes for a large extent of all learning in the workplace [2]. An example of informal learning might be that an employee faces a new issue at work. The employee recognizes the problem first and reflects on possible solutions. According to the results of the reflection, the employee might take certain actions. For example, the employee might search the internet or watch some videos to find solutions. The employee might then come up with a new solution, and discuss this with colleagues and the supervisor. Based upon their feedback, the employee might reflect how to optimize the solution, and then share the knowledge.

Thus, employees’ learn through different informal learning behaviors that predominantly are intentional and learner-controlled [3], such as keeping up-to-date or sharing knowledge [4].

However, the example above can also be viewed from the perspective of self-regulated learning (SRL) [5, 6]. SRL is one of the major conceptual frameworks at educational psychology, which includes cognitive, metacognitive, behavioral, motivational, and affective aspects of learning [5]. SRL provides an umbrella to study different variables such as learning strategies and how they contribute together to learning in a holistic and comprehensive way, which is described by various models, with different foci [5]. The ability to effectively learn self-regulated is a major competence to learn successfully [5]. However, learners may have deficits in SRL, such as not knowing strategies (so-called mediation deficit [7]), which can yet be successfully fostered by well-designed interventions (e.g., [8]).

According to a SRL perspective, the employee in the example above applies various learning strategies, which are defined by different SRL models, which aim to describe and understand successful learning [e.g., 5, 6]. For example, the task of searching the internet implies that the employee applies cognitive SRL learning strategies to search information to solve the problem. The employee might monitor whether the gathered information might be sufficient to reach the goal to find a satisfying new solution, which is a metacognitive SRL strategy [9]. Consequently, one could suggest, that informal learning behaviors might go along with different SRL strategies because they address partly similar behaviors. However, empirically, SRL assumptions have mainly been investigated in formal learning contexts such as school [e.g., 8, 10], university [e.g., 11], or professional development [e.g., 12]. In contrast, the role of SRL in informal workplace learning remains uncertain and studies are rare [13], although there are claims to integrate a “learning competence” [14] into models of informal learning. Learning competence is described as a set of various cognitive and metacognitive skills, such as “assessing specific learning needs, monitoring learning progress, and engaging in critical reflection” [14], which can be viewn as a a description of SRL learning strategies.

Further, research is lacking on links between SRL and informal learning strategies [15]. Therefore, this study aimed to investigate the relationship between informal learning strategies and SRL strategies in informal workplace learning. In this way, the study contributes to a better theoretical understanding of the connection of SRL and informal learning, thus linking SRL as one major research area in educational psychology with informal learning as main form of organizational learning [2], particularly with regards to strategies.

### Self-regulated learning

SRL is central to successful learning [e.g., 5, 6]. It is defined as “the modulation of affective, cognitive, and behavioral processes throughout a learning experience to reach a desired level of achievement.” [12]. In order to understand what behavior SRL consists of the example above might be viewed from the SRL perspective as described by the models of Zimmerman [6] or Schmitz and Wiese [16]. In SRL terms, the employee is motivated to learn how the specific problem might be solved. In the *forethought* or *preaction* phase, the employee analyzes the problem and plans how to achieve it successfully, i.e., by setting the goal to find a new solution. Further, the employee uses metacognitive planning strategies to plan how to reach the goal. Then, in the *performance* or *action* phase, the planned learning strategies are applied, which are in this example cognitive, i.e., searching the internet. Further, the process is monitored, which might result that the employee is unsatisfied whether the current solutions are enough to reach the goal, so searches for feedback, which might be viewed as a resource strategy in order to regulate metacognitively in SRL terms. Then the employee might apply further learning strategies to reach the desired outcome. Finally, the employee might reflect on the learning outcome, i.e., the solution reached, in the *self-reflection* or *postaction* phase and decides that this is satisfying and consequently shares it with colleagues.

In this example, the three phases of SRL described by Zimmerman’s and Schmitz and Wiese’s models are apparent. First, the learning process is initiated by motivation to learn and followed by a plan to achieve the learning goal, then the learners monitors if the chosen goal-directed strategies are sufficient and then reflects the learning process. Crucial to this process are, learning strategies, which describe the learner’s goal-directed affective, cognitive and behavioral processes and behaviors when learning self-regulated. As described above, learning strategies include cognitive, metacognitive, and resource strategies [9]. *Cognitive strategies* deal with the learning materials, and range from rather shallow rehearsing to deep-processing strategies of organization or elaboration. *metacognitive strategies* include planning, monitoring, and regulation, while *resource strategies* are centered on external resources like the learning environment or help-seeking behaviors [6, 17].

Whereas the assumptions of SRL have primarily been investigated in formal learning contexts such as school [8, 10], university [11], or professional development [12], the role of SRL in informal workplace learning remains uncertain due to the scarcity of relevant research [13]. Moreover, the overall links between SRL and informal learning strategies have attracted insufficient scholarly attention to date [15]. Accordingly, this study aimed to investigate the relationship between informal learning behaviors and SRL strategies in the workplace and build knowledge of how they are linked.

### Informal learning in the workplace

Informal learning behaviors are crucial for effective workplace learning. Employees learn to a large extent through informal learning behaviors [e.g., 2, 18] because formal training cannot provide all individually relevant training content in a fast-changing digitalized business world. Informal learning is usually defined in relation to formal learning on five continua, which show that informal learning behaviors usually have a low degree of structure, no external validation, no classroom setting, high learner control, and an internal learning stimulus [3]. More precisely:

Informal learning behaviors are non-curricular behaviors and activities pursued in service of knowledge and skill acquisition that take place outside formally-designated learning contexts. Such activities are predominantly self-directed, intentional, and fieldbased. Informal learning behaviors are not syllabus based, discrete, or linear

(Cerasoli et al., 2018, p. 204) [2].

Prominent models of informal learning is described by different models, from which prominent models are the model of include those of Tannenbaum et al. [19] and the recent model of Decius et al. [20]. Tannenbaum et al. [19] whose model underlies the above description of informal learning [2], describes that informal learning constitutes from four components: intent to learn, experience / action, feedback and reflection. These are facilitated by individual, organizational, and contextual factors. Employees can enter the process at any point and can experience one or more components once or on multiple occasions. However, the occurrence of all components is expected to be most effective for successful informal learning [19].

Decius et al.’s octagon model [20] added a second-order structure consisting of two factors for each of Tannenbaum et al.’s components [19]. In their model, intent to learn is divided into “extrinsic” and “intrinsic” intent to learn; experience / action is split into “trying and applying own ideas” and “model learning”; feedback consists of “direct” and “vicarious” feedback; and reflection consists of “anticipatory” and “subsequent” reflection. In addition, further models such as the one of Noe et al. [18] describe informal learning. Noe distinguished learning from oneself, from others and from non-interpersonal resources [18]. However, in contrast to the definition above, Noe et al.’s model also includes not only intentional learning, but also implicit actions. Yet, we want to focus on intentional learning behaviors, which are similar to self-regulated learning and align more closely with Cerasoli et al.’s view on informal learning, which is based on Tannenbaum et al.’s model and in line with Decius et al.’s model.

But how does informal learning work on a behavioral level? What behaviors do employees use? In order to examine how SRL and informal learning are related, informal learning behaviors are described next.

### Informal learning behaviors

From the various available descriptions of informal learning behaviors, we selected Bednall and Sanders [4] due to its strong behavior-orientation and consistency with the models described in the previous subsection. Bednall and Sanders [4] identified five informal learning behaviors: reflection, keeping up-to-date, feedback-seeking, knowledge sharing, and innovative behavior [4]. The first two are autonomous learning activities, whereas the latter three are collaborative.

The autonomous activity of *reflection on daily activities* encompasses recognizing one’s strengths and areas for development, checking progress toward goals, and changing one’s behavior to overcome perceived obstacles [4]. These behaviors fit with the “anticipatory” and “subsequent” reflections on past behavior described by Decius et al. [20] and is also similar to learning from oneself by Noe et al. [18]. Changing dissatisfaction with previous solutions would arise in response to the “experience and action” component of Tannenbaum et al.’s model [19]. The second autonomous behavior, *keeping up-to-date* refers to reading current articles or literature to keep up with current developments [4]. While it appears in Noe et al.’s framework [18], keeping up to date does not feature in Decius et al.’s model [20], despite its potential relevance to a range of informal and solution-focused learning behaviors.

Turning to the three collaborative learning behaviors, *feedback-seeking* refers to asking colleagues or supervisors for informal feedback and is part of Tannenbaum et al.’s [19] and Decius et al.’s [20] models. *Knowledge sharing* refers to the practice of exchanging information and skills with colleagues. Although it does not feature in either of the above models, knowledge sharing is key aspect of informal learning, as it empowers the informal learning of individuals in organizational networks. Lastly, *innovative behavior* encompasses finding novel, innovative solutions to problems and convincing colleagues to implement these [4]. Again, while this lies outside Tannenbaum et al.’s and Decius et al.’ models, trying and applying new ideas can be viewed as aspects of experience and action. In the example, the employee’s innovative problem solution is resulting of keeping up-to-date, feedback-seeking and reflecting. Moreover, the result of innovative behavior, innovation, might be possibly viewn as the ideal result of informal learning: new and innovative solutions, which accompany higher individual and corporate performance.

The goal of this current study is to analyze in which way these informal learning behaviors relate to learning strategies.

### Relationship between informal learning behaviors and self-regulated learning strategies

In general, learner control is a key aspect of informal learning [3, 18], which is typically described as self-directed and intentional [2]. The same applies to SRL in general and SRL strategies, which are intentional, deliberate, and goal-oriented actions. Both, self-regulated learning strategies and informal learning behaviors contribute to successful learning. Yet, at the level of in-depth behavior, it remains uncertain whether and in which way informal learning behaviors are related with SRL strategies?

One important informal learning behavior is *reflection on daily activities*. In the example, the employee contemplates the need for a new solution by reflecting on past behavior, which might be based upon experience and actions, and also use anticipatory reflection [19] to figure out the potential operation of a solution.

There appear to be links between anticipatory reflection and the metacognitive SRL strategy of planning, where learners plan how to reach a learning goal, part of the “forethought” phase in Zimmerman’s model [6], meaning in the example above to find a solution for the problem. On the other hand, reflecting on past behavior might accompany the metacognitive SRL strategy of monitoring, while behavioral changes arising due to experience and action [19] appear linked to the metacognitive SRL strategy of regulation. Employees may change their behavior, by deploying SRL deep-processing or resource strategies; for example, by elaborating on how they achieved previous goals [6]. Consequently, we anticipated that the informal learning behavior reflection would be linked to with (meta)cognitive SRL strategies in addition to resource strategies.

As an informal learning behavior, *keeping up-to-date*, entails continuously seeking information to solve problems [4]. In SRL terms, that previous metacognitive processes help employees to realize in the “performance” phase of the Zimmerman model [6] the need for more information. Subsequently, deep-processing SRL strategies, such as organization or elaboration strategies, are applied to organize the information that is found or connect it with previous topics [9]. We therefore anticipated that the informal learning behavior of keeping up-to-date would also align with metacognitive SRL strategies and cognitive SRL strategies.

*Feedback-seeking* is a collaborative informal learning behavior [4] that can be used to improve solutions to problems. According to the octagon model, feedback-seeking might be vicarious or direct and is one of the success factors of informal learning [20]. SRL models regard metacognitive monitoring strategies applied during the performance or reflection phase as a prerequisite of feedback-seeking since they uncover dissatisfaction with current solutions to problems [6]. Working toward the learning goal, an effective solution, involves applying the metacognitive strategy of regulation, and asking for feedback is a form of help-seeking, a SRL resource strategy [6]. Consequently, we predicted that feedback-seeking would be an informal learning behavior that co-occurs with both SRL metacognitive and resource strategies.

*Knowledge sharing*, might be defined as “model learning” in the octagon model [20]. In Tannenbaum et al.’s [19] terms it could also be viewed as as a trigger event for informal learning as colleagues test new solutions via experience and action. To successfully share knowledge, employees must analyze the knowledge or skills to be shared, in terms of content, recipients, and timing. This behavior is described as the metacognitive strategy of planning in the “forethought” phase of Zimmerman’s model [6]. For example, should the knowledge be shared in person, in a virtual meeting, or on the company blog? The sharer might have the individual goal of learning something by discussing a particular aspect of the shared knowledge, which might result in an individual learning goal in Zimmerman’s model [6]. When sharing knowledge, to convey shared knowledge appropriately, it should be organized and described comprehensibly, by finding analogies or connections to other topics, for instance, which might be described as deep-processing SRL strategies [6]. Knowledge sharing may therefore imply the deep-processing SRL strategies of organization and elaboration as well as that of monitoring, since analyzing the likely recipients of one’s knowledge also entails considering its value to others [6]. As an informal learning behavior, knowledge sharing also demands additional effort, indicating its link to the SRL resource learning strategy of effort regulation. Consequently, as an informal learning behavior, knowledge sharing is expected to occur alongside with SRL (meta)cognitive and resource strategies.

Lastly, *innovative behavior* draws on experience and action (Tannenbaum et al.’s model, [19]) as well as further informal learning behaviors to produce novel solutions. In the SRL terms of Zimmerman’s model [6], the employee must first entertain the forethought that a new solution is required, indicating the potential link between innovative behavior and the metacognitive SRL strategy of planning. During the performance phase in Zimmerman’s SRL model [6], different strategies might enable the learning goal of innovating a solution to be reached: Employees could use deep-processing SRL strategies to develop the level of understanding needed to find new solutions. If their current approach does not achieve their goal, they could use metacognitive monitoring and regulation strategies and ask colleagues for an SRL resource strategy. Striving to find and communicate new solutions to problems utilizes the SRL resource strategy of regulating effort. Overall, innovative behavior occurs alongside with SRL (meta)cognitive and resource strategies.

This comparison has focused on SRL strategies and the self-regulation process itself, while paying less attention to aspects of SRL such as emotions [5], which current conceptualizations of informal learning have not fully explored [2, 14]. As the foregoing description shows, many possible relationships and overlaps exist between informal learning behaviors and SRL strategies in the workplace. Intuitively, informal learning behavior appears be closely linked to SRL strategies, but empirical results demonstrating this linkage are rare. A few previous studies have investigated SRL in informal workplace learning, supporting the relevance of SRL strategies for successful informal workplace learning, yet, that research did not extend to investigate the relation between SRL strategies and informal learning behaviors [13, 21, 22]. Nonetheless, several studies point to the importance of motivation in terms of goal orientation, motivation to learn, and self-efficacy as a prerequisite for successful informal learning [2]. These relationships are relevant because motivation is also a necessary condition of successful SRL [6].

### Summary of research questions

**Fig 1:**
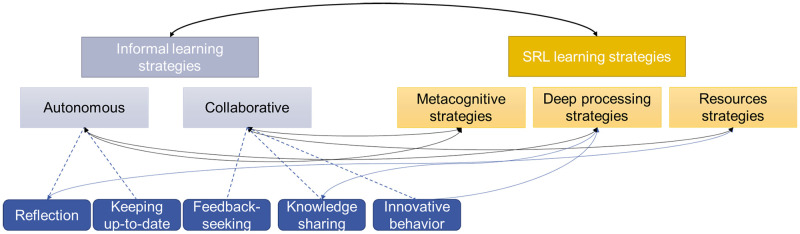
The expected relationship between informal learning behaviors and self-regulated learning (SRL) strategies.

This study investigates the relationship between informal learning behaviors and SRL in detail. In general, we anticipated that informal learning behaviors and SRL strategies would be positively related (Hypothesis 1; Fig 1). We also predicted the presence of a positive relationship between the autonomous informal learning strategies of reflection and keeping up-to-date with the metacognitive SRL strategies of planning, monitoring, and regulation (Hypothesis 2). Specifically, we hypothesized that reflection, an autonomous informal learning behavior, would be positively associated with deep-processing and resource SRL strategies (Hypothesis 2a). We also anticipated that the autonomous informal learning behavior of keeping up-to-date, would be positive related with deep-processing SRL strategies (Hypothesis 2b). Lastly, we hypothesized a positive relationship between the collaborative informal learning behaviors of feedback-seeking, sharing knowledge, and innovative behavior with the metacognitive and resource-based SRL strategies (Hypothesis 3). We assumed that knowledge sharing (3a) and innovative behavior (3b), would positively correlate with deep-processing SRL strategies (Hypotheses 3a and 3b).

## Materials and methods

### Participants

We recruited 248 participants who met the requirement of current employment, i.e., working full time or part-time (the prerequisite was that the participants work at least 20 hours a week). Participants’ age ranged widely (*M* = 42.51 years, range 16–84 years), and 61% were female. Most participants were able to look back on several years of work experience (*M* = 17.35, *SD* = 14.22) and worked full-time (*M* = 36.5 hours per week, *SD* = 9.98). Participants were well-qualified, with 61% having a university degree followed by 20% who had undertaken vocational training. They worked in companies, mainly in the industrial sector (20%) followed by the service sector (19%).

### Procedure

Participants were invited to the LidA project channels to participate. The LidA project “Lernen in der digitalisierten Arbeitswelt” [Learning in the digitized working world] is a consortium project with diverse company partners in German industry. The LidA project deals with the effects of digital transformation. The central question is how company and employee-specific teaching and learning modules can enable employees to meet the challenges of digital change.

Data were obtained using online questionnaires embedded at the beginning of digital learning opportunities provided by the LidA project on the ILIAS learning management system and SoSci survey. ILIAS learning management system is a learning management system, which allows to embed surveys. The participants registered for the learning opportunities provided for interdisciplinary digital competencies by the LidA project, which were shared internally in the large German industrial and automotive companies, which participated in the LidA project, and received access to ILIAS. The survey was embedded at the beginning of the learning opportunities, and the participants took part in it pseudo-anonymously. The participants received a certificate if they completed the complete learning opportunity. If the participants did not log in to ILIAS they received a reminder after two weeks, and if they registered but did not participate they also received up to three reminders every two weeks. Participation was voluntary, and all participants signed an informed consent form before the survey. Following German legislation, institutional review board approval was not required for this type of study. This study complies with human subjects guidelines of national research committees and the APA Ethics Code Standards.

### Measures

We used established self-report likert-scales to assess SRL and informal learning activities in the workplace. Table 1 shows confirmatory factor analysis (CFA) for the measured scales and see S1 Data for an overview for the used items.

**Table 1:** Results of confirmatory factor analysis for SRL and informal learning strategies.

Scale	CFA result	Model comparison
Planning	χ^2^(5) = 16.917, CFI = 0.924, RMSEA (90% CI) = 0.105 [0.058; 0.155], SRMR = 0.051	AIC: 2413; BIC: 2464
Monitoring and regulation	χ^2^(5) = 5.176, CFI = 0.999, RMSEA (90% CI) = 0.013 [0.000; 0.097], SRMR = 0.023	AIC: 2431; BIC: 2482
Elaboration	χ^2^(2) = 1.618, CFI = 1.000, RMSEA (90% CI) = 0.000 [0.000; 0.107], SRMR = 0.014	AIC: 1520; BIC: 1561
Help-seeking	Saturated model	
Effort regulation	Saturated model	
Help-seeking correlated with effort regulation	χ^2^(4) = 1.228, CFI = 1.000, RMSEA (90% CI) = 0.000 [0.000; 0.055], SRMR = 0.021	AIC: 2001; BIC: 2056
Reflection	Saturated model	
Keeping up-to-date	Saturated model	
Reflection correlated with keeping up-to-date	χ^2^(8) = 17.536, CFI = 0.975, RMSEA (90% CI) = 0.074 [0.026; 0.120], SRMR = 0.034	AIC: 3429; BIC: 3493
Feedback-seeking	Convergence problems ^a^	
Knowledge sharing	Saturated model	
Innovative behavior	Convergence problems ^a^	
Feedback-seeking correlated with knowledge sharing and innovative behavior	Convergence problems ^a^	
Reflection correlated with feedback-seeking and knowledge sharing	χ^2^(74) = 145.482, CFI = 0.858, RMSEA (90% CI) = 0.067 [0.050; 0.080], SRMR = 0.094	AIC: 3592; BIC: 3744
Reflection correlated with feedback-seeking with 8 items ^b^ and knowledge sharing	χ^2^(62) = 103.388, CFI = 0.910, RMSEA (90% CI) = 0.055 [0.036; 0.074], SRMR = 0.084	AIC: 3394; BIC: 3536
Reflection correlated with innovative behavior	χ^2^(45) = 448.998, CFI = 0.978, RMSEA (90% CI) = 0.036 [0.000; 0.065], SRMR = 0.052	AIC: 2750; BIC: 2855

*Note*.

^a^ Due to convergence problems with the collaborative informal learning strategy factors of feedback-seeking and innovative behavior, models correlated with the autonomous informal learning strategy of factor reflection were estimated because it is theoretically assumed that autonomous and collaborative strategies are correlated [4].

^b^ One item, “If I think I’ve done my job poorly, I discuss it with my manager,” was excluded due to low factor loading and because it targeted a slightly different topic, i.e., error or management culture, and not primary feedback-seeking. Due to better model fit, the eight-factor solution was used in subsequent analyses.

### Self-regulated learning

We used Kittel et al.’s [13] measure, adjusted for informal workplace learning to assess SRL strategies with 19 items (Cronbach’s α = .884). The measure is based on the MSLQ questionnaire [23], Pintrich’s learning strategy taxonomy [17], and SRL models [5]. Adjustments were made for the involved people (e.g., colleague instead of student), the setting (work instead of class), and materials (work materials instead of study materials). Formal learning aspects were excluded, such as learning for tests, but general work settings and situations, such as meetings, were included. Two to four items each measured the metacognitive strategies of planning (α = .717), monitoring and regulation (α = .726), the deep-processing cognitive strategy of elaboration (α = .748), and the resource strategies of help-seeking (α = .541) and effort regulation (α = .707).

### Informal learning

To measure informal workplace learning strategies, we used Noe and colleagues’ [18] measure to evaluate learning from oneself, i.e., reflection (α = .667), and from non-intrapersonal resources, i.e., keeping up-to-date (α = .836) with three items each. We did not use the scale learning from others because it targets only at the interaction with others but not on feedback-seeking. We measured the collaborative learning activity of feedback-seeking with eight items (α = .881) [24]. Knowledge sharing was measured with two items (α = .834) [25, 26] and innovative behavior (α = .917) with seven items [27].

### Rationale for analyses

First, we checked the data for outliers and irregularities. Next, we applied CFA to analyze the model fit of the respective factors by using the lavaan package in R [28]. Structural equation modeling (SEM) was used to answer the research questions. We estimated models for each hypothesis according to expectations and then reduced them to less complex models. Finally, we used model comparisons of the nested models to identify the model that describes the data best.

## Results

### Preliminary analyses

To begin with, we analyzed the descriptive data and correlations (Table 2). Correlations suggested a strong relationship between informal learning behaviors and SRL strategies. For informal learning in general, there was a strong relation with all SRL strategies except for help-seeking. For some informal learning strategies, there was a strong relationship between monitoring and regulation and a moderate relationship with planning. A strong correlation was found between effort regulation for knowledge sharing and innovative behavior.

**Table 2:** Means, standard deviations, and correlations between study variables.

Variables	*MW* (*SD*)	1.	2.	3.	4.	5.	6.	7.	8.	9.	10.	11.	12.
1. Planning	3.99 (.55)	-	.469***	.295***	.264***	.497***	.761***	.184**	.172*	.194	.296**	.370**	.205**
2. Monitoring & regulation	3.73 (.55)		-	.417***	.241***	.503***	.784***	.443***	.308***	.268*	.364**	.451***	.424***
3. Elaboration	4.45 (.50)			-	.261***	.556***	.660***	.284***	.208**	.282*	.301**	.377***	.302***
4. Help-seeking	3.91 (.66)				-	.383***	.579***	.132	-.070	.233*	.315**	.163	.035
5. Effort regulation	4.27 (.68)					-	.712***	.144	.282*	.262*	.365**	.441***	.394***
6. SRL strategies	4.01 (.40)						-	.367***	.229***	.317**	.422***	.472***	.359***
7. Reflection	3.16 (.73)							-	.310***	.382***	.385***	.620***	.665***
8. Keeping up-to-date	3.20 (1.01)								-	.376***	.357**	.549***	.799***
9. Feedback- seeking	2.88 (.74)									-	.624***	.469***	.813***
10. Knowledge sharing	3.62 (.97)										-	474***	.704***
11. Innovative behavior	3.71 (.76)											-	.840***
12. Informal learning behaviors	3.26 (.64)												-

*Note*.

* *p* < .05,

** *p* < .01,

*** *p* < .001.

### Relationship between informal learning strategies and self-regulated learning strategies

SEMs were applied to determine how informal learning strategies and SRL strategies are related.

**Fig 2:**
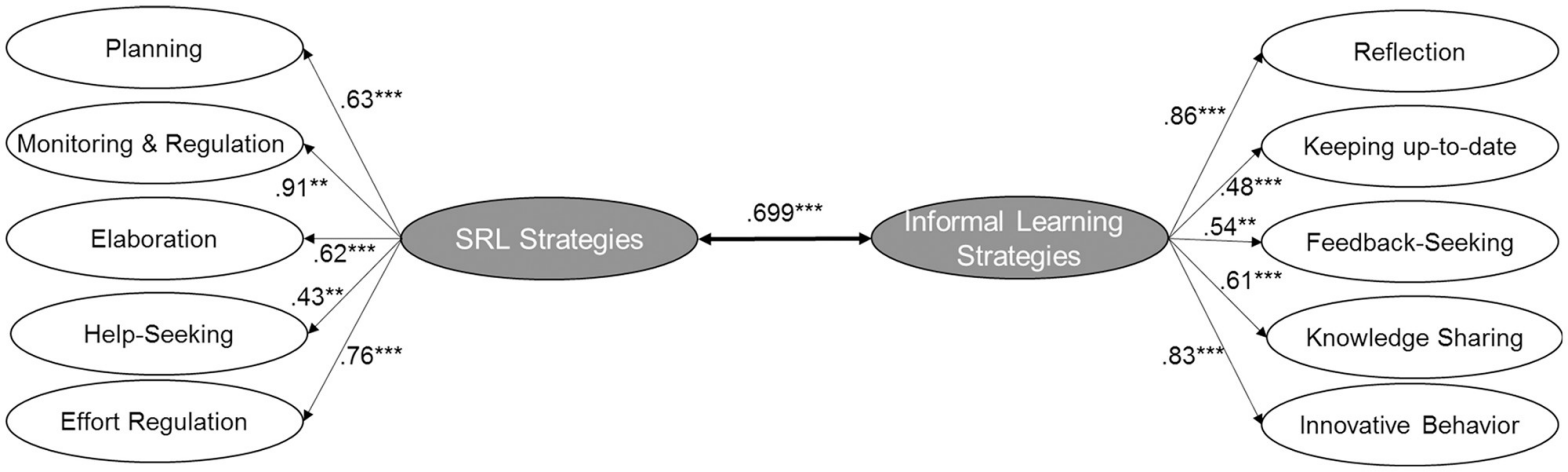
Structural equation model showing the relationship between SRL strategies and informal learning strategies. Coefficient is significant at the .05 level *, at the .01 level ** and at the .001 level ***. *N* = 248. The model fit is χ^2^(862) = 3464.522, CFI = .735, RMSEA (90% CI) = .063 [.058; .068], SRMR = .117.

To evaluate the first hypothesis regarding the relationship between SRL strategy use and informal learning behaviors in general, we estimated an SEM with two correlated latent factors—SRL strategies and informal learning strategies. The two latent factors were strongly correlated (ρ = .699, *p* < .001; Fig 2), supporting the first hypothesis. However, the model fit was not satisfactory, suggesting that the expected informal learning strategy factor structure with a latent factor and the strategies as subfactors was not completely accurate. A more complex factor structure for strategy level should be more accurate. As a consequence, to analyze the subsequent hypotheses, we estimated the models separately for each informal learning strategy.

### Autonomous learning strategies and self-regulated learning strategies

To address the second hypothesis regarding the relationship between autonomous informal learning strategies and SRL strategies, we compared several SEM models (Table 3). Models with the expected relationships were compared with less complex models. We used model comparison to determine which model best describes the data. Results showed that less complex models described data better than models including more SRL strategies. A model that connects reflection and keeping up-to-date with only monitoring and regulation described the data significantly better than a model that included planning, elaboration, and resource strategies. Additionally, in the more complex models 1a and 2a (Table 3), the informal learning behaviors were only significantly predicted by the use of monitoring and regulation (e.g., reflection: β_monitoring and regulation_ = .672, *p* = < .001, keeping up-to-date: β_monitoring and regulation_ = .378, *p* = < .001). Consequently, these results only partly support hypotheses 2a and 2b.

**Table 3:** Results of structural equation modeling of the relationship between informal learning activities and self-regulated learning (SRL) strategies.

Model	SEM result	Significant predictor of informal learning behavior	Model comparison to more complex model
Autonomous behaviors			
Model 1a: reflection predicted by metacognitive, cognitive, and resources SRL strategies	χ^2^(194) = 298.73, CFI = 0.902, RMSEA (90% CI) = 0.050 [0.038; 0.060], SRMR = 0.067	Monitoring and regulation: .727, *p* = < .001	AIC = 9856; BIC = 10131
Model 1b: reflection predicted by monitoring and regulation	χ^2^(19) = 19.76, CFI = 0.998, RMSEA (90% CI) = 0.013 [0.000; 0.059], SRMR = 0.034	Monitoring and regulation: .672, *p* = < .001	AIC = 4052; BIC = 4136 Model comparison to Model 1a Δ χ^2^(175) = 279.63, *p* < .001
Model 2a: keeping up-to-date predicted by metacognitive and cognitive SRL strategies	χ^2^(113) = 176.91, CFI = 0.936, RMSEA (90% CI) = 0.051 [0.0368; 0.0604], SRMR = 0.060	Monitoring and regulation: .400, *p* = .001	AIC = 8036; BIC = 8229
Model 2b: keeping up-to-date predicted by monitoring and regulation	χ^2^(19) = 15.59, CFI = 1.00, RMSEA (90% CI) = 0.00 [0.000; 0.046], SRMR = 0.029	Monitoring and regulation: .378, *p* = < .001	AIC = 4186; BIC = 4270 Model comparison to Model 2a Δ χ^2^(94) = 161.3, *p* < .001
Collaborative behaviors and innovative behavior			
Model 3a: Feedback-seeking predicted by metacognitive and resources SRL strategies	χ^2^(220) = 320.63, CFI = 0.882, RMSEA (90% CI) = 0.046 [0.034; 0.056], SRMR = 0.088	Monitoring and regulation: .300, *p* = .129	AIC = 8158; BIC = 8426
Model 3b: Feedback-seeking predicted by monitoring and regulation	χ^2^(64) = 86.16, CFI = 0.948, RMSEA (90% CI) = 0.040 [0.011; 0.060], SRMR = 0.064	Monitoring and regulation: .317, *p* = .056	AIC = 3813; BIC = 3949 Model comparison to Model 3a Δ χ^2^(156) = 234.32, *p* < .001
Model 4a: Knowledge sharing predicted by metacognitive, cognitive, and resources SRL strategies	χ^2^(174) = 283.03, CFI = 0.894, RMSEA (90% CI) = 0.053 [0.042; 0.064], SRMR = 0.080	Planning: .457, *p* = .022	AIC = 8601; BIC = 8866
Model 4b: Knowledge sharing predicted by planning	χ^2^(13) = 22.16, CFI = 0.964, RMSEA (90% CI) = 0.057 [0.007; 0.095], SRMR = 0.043	Planning: .269, *p* = < .001	AIC = 2275; BIC = 2850 Model comparison to Model 4a Δ χ^2^(161) = 260.77, *p* < .001
Model 5a: Innovative behavior predicted by metacognitive, cognitive, and resources SRL strategies	χ^2^(325) = 1581.847, CFI = 0.870, RMSEA (90% CI) = 0.052 [0.043; 0.061], SRMR = 0.087	Monitoring and regulation: .613, *p* = .026 effort regulation: .732 *p* = .101	AIC = 9333; BIC = 9353
Model 5b: Innovative behavior predicted by monitoring and regulation and effort regulation	χ^2^(91) = 611.629, CFI = 0.973, RMSEA (90% CI) = 0.030 [0.000; 0.051], SRMR = 0.064	Monitoring and regulation: .281, *p* = .235 effort regulation: .364, *p* = .139	AIC = 3842; BIC = 3995 Model comparison to Model 5a Δ χ^2^(210) = 367.54, *p* < .001

*Note*. Theoretically expected models (a) were compared with reduced models with empirically supported relations (b). Model comparison supported Model 1b, 2b, 3b, 4b, and 5b.

### Collaborative learning strategies and self-regulated learning strategies

**Fig 3:**
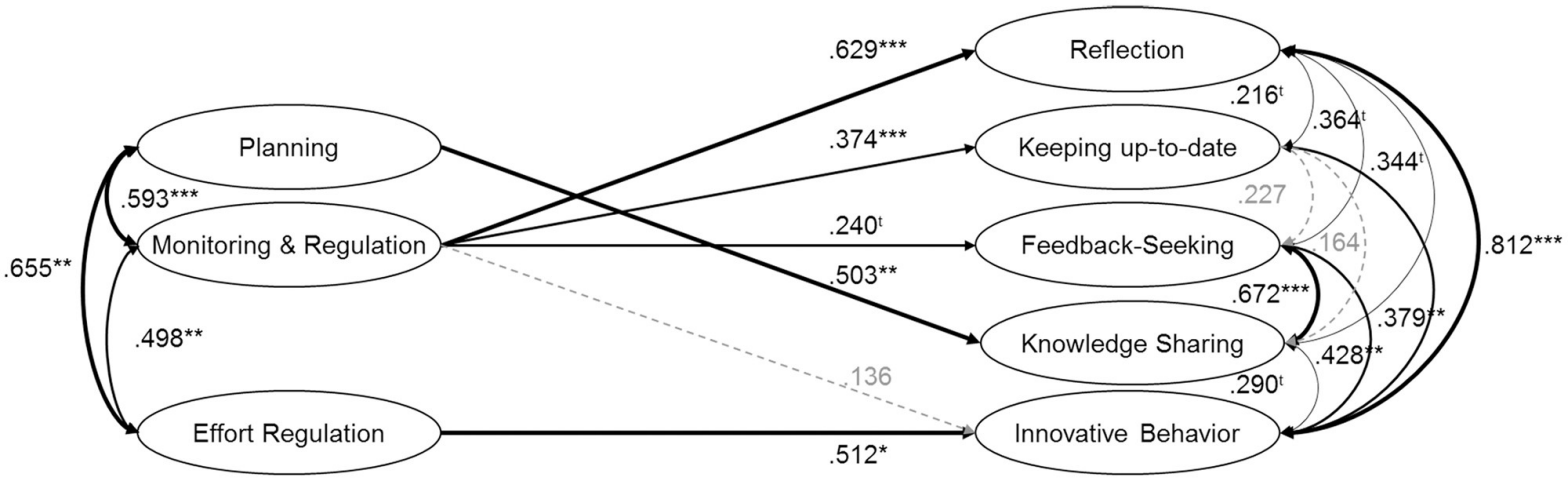
Structural equation model showing the relationship between SRL strategies and informal learning strategies. Coefficient is significant at the .10 level ^t^, at the .05 level *, at the .01 level ** and at the .001 level ***. Paths that are n.s. are gray-colored. *N* = 248. The model fit is χ^2^(595) = 2505.430, CFI = .823, RMSEA (90% CI) = .054 [.048; .061], SRMR = .097.

To investigate the third hypothesis—how the collaborative informal learning behaviors of feedback-seeking, sharing knowledge and innovative behavior relate to SRL strategies—SEM models were compared (Table 3). Results also supported the less complex models: feedback-seeking was only predicted by monitoring and regulation, whereas the metacognitive strategy of planning predicted knowledge sharing (β_Planning_ = .457, *p* = .022). Finally, innovative behavior was predicted by monitoring and regulation, and by effort regulation, but the results were inconsistent. Some of the models showed a greater influence of effort regulation (Fig 3; Table 3), while others showed monitoring and regulation had more impact (Table 3). Consequently, these results only partly support hypotheses 3a and b.

In sum, results suggest that autonomous and collaborative informal learning activities, except innovative behavior, are associated with the use of metacognitive SRL strategies. Innovative behavior is associated with effort regulation. Fig 3 shows the complete model.

## Discussion

This study investigated the relationship between informal learning behaviors and SRL strategies. The results showed that informal learning behaviors were linked to metacognitive strategies such as monitoring and regulation, while innovative behavior was associated with effort regulation. However, we found that informal learning behaviors were neither associated with deep-processing nor SRL resource strategies.

### Informal learning behaviors and self-regulated learning strategies

#### Autonomous behaviors

The autonomous behaviors of reflection and keeping up-to-date were, as expected, positively correlated with metacognitive SRL strategies, suggesting that employees reflected on past behavior or anticipated future behavior using metacognitive SRL strategies.

We assumed that employees reflecting on certain experiences and actions would use SRL monitoring strategies to check their own behavior during specific tasks. Surprisingly, however, reflective behaviors were correlated with monitoring and regulation, but not with planning. Thus, employees engaged in informal learning appeared only to apply metacognitive SRL strategies in the “performance” but not the “forethought” phase of Zimmerman’s model [6], indicating the potential benefits of focusing on the use of anticipatory reflection to strategically plan subsequent behavior. Future studies might investigate the effectiveness of interventions to foster metacognitive planning strategies for informal learning. Moreover, contrary to expectations, reflection was not associated with deep-processing and resource SRL strategies. This suggests that employees may reflect at superficial levels without elaborating on their previous experiences or drawing on their resources, despite the importance of these to successful SRL during the performance phase [6]. While using a wider range of reflective strategies could lead to positive behavioral changes, successful reflection is an advanced competence that might require further training [29]. Our results suggest that the informal learning behavior of reflection might require further development since it impacts the SRL process.

Employees try to keep up-to-date through monitoring and regulation strategies that help them identify gaps in their knowledge. However, our results suggested that staying updated was not linked to the strategies of organization or elaboration: employees may have a “mediation deficit” wherein they are either unaware of certain strategies or deliberately choose not to use them–, which is a so-called “production deficit” [7, 30, 31]. Thus, the employees may briefly address a gap in their knowledge by considering some new information, but not retaining this for later, more detailed review. This may explain why models of informal learning, such as Tannenbaum et al. [19] do not regard keeping up-to-date as informal learning behavior. However, we suggest that searching for additional information is a crucial strategy that would supplement the informal learning behaviors described in Tannenbaum et al.’s [19] or Decius et al’ models [20]. Future studies might investigate how training to help employees organize information might help them keep up-to-date more effectively, for example.

#### Collaborative behaviors

As expected, the collaborative behaviors of feedback-seeking and knowledge sharing were associated with various metacognitive strategies, whereas innovative behavior was linked to the resource strategy of effort regulation. Feedback-seeking was associated with monitoring and regulation but not with the resource strategies of seeking help or regulating effort. The employees thus appeared to seek feedback during Zimmerman’s “performance” phase [6] as a result of monitoring and regulation although these latter strategies may have been a response to the feedback they received. However, employees may potentially learn more from feedback by using further SRL resource strategies, such as planning in forethought [6] to think through possible informal learning behaviors.

The link found between sharing knowledge and planning did not extend to resource strategies. While the employees planned when to share their own knowledge, they did not appear to interpret this as an opportunity to increase their knowledge by seeking help. Surprisingly, knowledge sharing was not associated with metacognitive strategies such as monitoring and regulation, suggesting that employees might not reflect when sharing information with others. The employees seemed to use planning strategies during the “forethought” phase of SRL, but perhaps did not set themselves learning goals and therefore did not subsequently use learning strategies of “performance” or “self-reflection” phases [6]. Given the increasing importance of knowledge sharing in a fast-changing world, training employees in this skill might increase its benefits in the workplace.

Finally, innovative behavior, i.e., finding new and innovative solutions for problems, was associated with the regulation of effort but not with deep processing, metacognitive strategies, or help-seeking. Previous studies have demonstrated the importance of investing effort into problem solving [e.g., 32]. Effort can be invested to support learning, but it is also possible that employees might selectively focus on investing into particular aspects of tasks, such as understanding materials. The lack of relationship with the other learning strategies suggested that the employees might have focused wholly on innovation while overlooking potential resources, such as colleagues, that could be identified through metacognitive strategies or elaboration from previous experiences. The results imply that employees may not try to learn intentionally when innovating and might consequently not initiate a SRL process. That is to say, employees may not view attempts to find new and innovative solutions as opportunities to learn.

Prominent models of informal learning (Tannenbaum et al. [19]; Decius et al. [20]; Noe et al. [18]) do not include innovative behavior and therefore do not view it as a learning opportunity, a notion supported by the absence of relationships with SRL strategies in the present study. On the other hand, results showed that innovative behavior does occur alongside informal learning behaviors, which supports that it could be inserted into existing models. A previous study further supports an integration into models by showing relations between innovative behavior and collaborative informal learning behaviors, i.e., acting upon feedback from colleagues as well as information seeking from others, and innovation-specific reflection [33]. Innovative behavior was further distinguished into opportunity exploration, idea generation, idea promotion and idea realization [33]. Whereas reflection was strongly associated with all factors of innovative behaviors, collaborative informal learning behaviors were only weakly associated with opportunity exploration, idea generation and idea promotion, but not with idea realization [33]. This is in line with the results of the present study, which show as well the strong relation between innovative behavior and reflection, but also link innovative behavior with collaborative informal learning behaviors and keeping up-to-date. As a consequence, collaborative informal learning behaviors help employees to generate innovative ideas and promote them but individual reflection is necessary to actually realize the idea. Consequently, models of informal learning might include innovative behavior, linked with collaborative behaviors, reflection and keeping up-to-date and, in addition, should emphasize the role of effort.

The results of this study indicate that employees may use only a few metacognitive SRL strategies during informal workplace learning. This finding is in line with a previous study, which shows that individuals with higher metacognitive skills are more likely to engage in informal learning behaviors and transfer their acquired knowledge [34]. However, these results are contrary to the assumption that a general learning competence in terms of also cognitive strategies [14] is a prerequisite for all informal learning behaviors. Consequently, there may be a mediation or production deficit of SRL strategies. Staff members who integrate further SRL strategies into their informal learning behaviors may learn more effectively, benefitting themselves, their colleagues, and their organizations [2]. Our findings indicate the need to foster the use of more effective informal learning strategies in the workplace.

### Informal and self-regulated learning

The results highlighted a strong relationship between informal learning and SRL, raising the question about their causal dependence and separateness as constructs. If separable, self-regulated learning ability, particularly of metacognitive skills, may be the necessary prerequisite for effective informal learning. However, the results also demonstrated that the employees did not associate all SRL skills with informal learning, particularly cognitive strategies such as organization or elaboration, or resource strategies such as seeking help. This suggests a potential mediation or production deficit, wherein employees are unaware of these strategies or unwilling to apply them [7, 30, 31]. Thus, subsequent research might investigate how SRL enables informal learning or vice versa. Do informal learning behaviors encourage SRL within the workplace or vice versa? Alternatively, do items to measure SRL strategies and informal learning behaviors overlap? They may represent a so-called jangle fallacy, where tests claim to measure different constructs but actually assess the same entity [35]. An approach for analyzing this question might be an analysis of discriminant and convergent validity. An approach to analyze the discriminant and convergent is applying a so-called multitrait-multimethod analysis, which was first proposed by Campbell and Fiske [36] (see for examples [37, 38]). A multitrait-multimethod analysis distinguished between shared variance due to traits vs. shared variance due to methods [36].

Similarities also arise when considering the antecedents and outcomes of SRL and informal learning: both informal learning behaviors and SRL strategies are linked to personal antecedents such as motivation or self-efficacy [2, 12]. However, existing theoretical models treat these variables differently because they are often part of SRL models [5], but are viewed as antecedents or outcomes of informal learning behaviors [2]. Fewer empirical studies of job-related antecedents such as job characteristics have been conducted, but the available results indicate they are positively, however indirectly related over motivation with SRL strategies [13]. Regarding informal learning behaviors, job-related antecedents are shown to support informal learning [2]. Both SRL and informal learning behaviors are positively associated with successful learning outcomes [2, 12, 39]. Interestingly, most SRL studies tend to focus more on academic vs. tacit knowledge, although SRL has been shown to positively impact attitudes and attributions [12]. Regarding job performance, there is currently a lack of evidence linking SRL to job performance whereas it is positively related to informal learning behaviors [2].

Although informal learning is defined as an intentional learning process outside formally designated learning contexts [2], some informal learning processes may be reactive rather than purely intentional [40]. More effective informal learning may occur when employees are encouraged to use strategies to learn something new. For example, companies could include the presentation of the results of informal learning into team meetings to highlight the value of such learning to organizations.

### Theoretical implications

In this study, we described how Bednall and Sanders’ [4] classification can be partly integrated into models and frameworks of informal learning [e.g., 18] and is comparable with other frameworks [2]. Our results suggest that Bednall and Sanders’ classification [4] of workplace behavior into “autonomous” and “collaborative” types is not wholly accurate. Innovative behavior, in particular, seems to differ from other collaborative types of behavior because it focuses more strongly on problem-solving than other behaviors, as discussed above. Thus, a tripartite classification into “autonomous”, “collaborative”, and “innovative” behavior may make more sense.

The close link between informal learning behaviors and some SRL strategies shown in this study suggests that models of informal learning might be extended by adding metacognitive SRL strategies, in particular. As discussed above, our results also demonstrated the possibility of integrating innovative behavior and keeping up to date into models of informal learning, which would then need to be extended and re-tested.

It is important to underline that definitions of particular informal learning behaviors vary considerably. For example reflection occurs throughout the entire SRL process, while other behaviors such as feedback-seeking are more comparable with a single learning strategy in SRL. Consequently, a revision of informal learning behaviors is necessary. A new model could include informal learning behaviors and SRL strategies and consider their relationship. Further, strategies or behaviors that exert a trigger effect on further informal learning behaviors would be an important focus for future studies. For instance, feedback may trigger reflection, leading to goal setting and then to the use of further strategies to achieve the goal.

### Practical implications

In the workplace, intentional informal learning behaviors should be supported by managers and learning departments [2]: Employees could be encouraged to learn informally or provided with opportunities to share knowledge gained from informal learning. To achieve this goal, companies might strive for a more encouraging organizational learning culture, which is shown to be positively associated with the motivation to learn [13]. The organizational learning culture depends strongly on the leadership style [41], showing the crucial role of managers in informal learning [42]. In order to promote informal learning, manager cannot regulate directly informal learning due to its autonomous nature, but they can design an environment where employees feel secure to learn and share their knowledge, for example by providing social support to their employees, or by having managers exemplify a positive error culture [42]. In addition to that, companies can also help their managers by providing them with information about informal learning in general and how to promote informal learning behaviors (see a more comprehensive overview of manager recommendations at [42]). For a comprehensive list of possible antecedents, sorted into different categories, which companies can use to analyze their current informal learning environment and derive suitable support measures, see the review of Jeong and colleagues [14].

Beyond this, our results suggest that more effective and intentional learning behaviors must be promoted in the workplace. This could be achieved by learning strategy training to compensate for possible mediation or production deficits since earlier SRL studies have shown that training might increase the use of learning strategies [8].

### Limitations

Despite the diversity of our sample of participants in age, gender, and occupation, the voluntary basis of their participation may limit the generalizability of our results due to self-selection bias. An additional limitation on generalizability may be cultural; because only German company employees took part, the results may only be valid for populations from the cultural “West.”

Self-report measures may have led to further bias, but this approach to data collection did manage to capture relevant broad features of the constructs [43] of SRL and informal learning to obtain initial insights into their relationship. Research that takes a a more process-oriented approach is needed to understand the actual learning processes and more closely determine whether the nature of the relationship between SRL and informal learning was a prerequisite, the same or a subset, or behaviorial outcome. In addition, the results might be biased by common method bias as it might serve as another explanation for high correlation of constructs in cross-sectional self-report design [44].

We could not include all the factors from the SRL models [5], and these should be investigated in future studies. Moreover, we measured for SRL in informal workplace learning only, not in formal learning contexts, which limits our results to employees’s use of SRL and informal learning strategies in the workplace. Future research might explore how formal and informal strategies are associated. While there are preliminary indications that formal learning opportunities precede informal learning [4], it remains unclear whether this occurs directly or indirectly as a transfer effect between strategies. However, informal learning behaviors are very difficult to capture due to their nature. They do not have a defined start or endpoint, and process-oriented measures such as regular questions might invalidate observations by serving as prompts to participants.

Overall, this study has shown that informal learning and regulated learning ability are closely connected. Specifically, employees rely on the metacognitive strategies of monitoring and regulation when learning informally. However, employees seem to experience mediation or production deficits when executing informal learning behaviors that benefit from deep processing and resource strategies.

This is why we recommend that employees augment informal learning with additional resources that may boost their individual success and that of their organizations.

## Supporting information

S1 Data (SAV)
